# Intrauterine and Neonatal Exposure to Opioids: Toxicological, Clinical, and Medico-Legal Issues

**DOI:** 10.3390/toxics11010062

**Published:** 2023-01-09

**Authors:** Giuseppe Davide Albano, Corinne La Spina, Walter Pitingaro, Vanessa Milazzo, Valentina Triolo, Antonina Argo, Ginevra Malta, Stefania Zerbo

**Affiliations:** Section of Legal Medicine, Department of Health Promotion, Mother and Child Care, Internal Medicine and Medical Specialties, University of Palermo, 90129 Palermo, Italy

**Keywords:** intrauterine, neonatal, opioids, exposure, forensic toxicology, medico-legal issues

## Abstract

Opioids have a rapid transplacental passage (i.e., less than 60 min); furthermore, symptoms characterize the maternal and fetal withdrawal syndrome. Opioid withdrawal significantly impacts the fetus, inducing worse outcomes and a risk of mortality. Moreover, neonatal abstinence syndrome (NAS) follows the delivery, lasts up to 10 weeks, and requires intensive management. Therefore, the prevention and adequate management of NAS are relevant public health issues. This review aims to summarize the most updated evidence in the literature regarding toxicological, clinical, and forensic issues of intrauterine exposure to opioids to provide a multidisciplinary, evidence-based approach for managing such issues. Further research is required to standardize testing and to better understand the distribution of opioid derivatives in each specimen type, as well as the clinically relevant cutoff concentrations in quantitative testing results. A multidisciplinary approach is required, with obstetricians, pediatricians, nurses, forensic doctors and toxicologists, social workers, addiction specialists, and politicians all working together to implement social welfare and social services for the baby when needed. The healthcare system should encourage multidisciplinary activity in this field and direct suspected maternal and neonatal opioid intoxication cases to local referral centers.

## 1. Introduction

Heroin and methadone are the opioids most frequently used by pregnant women. It is estimated that approximately 7000 opiate-exposed births occur annually [1]. The degree of intrauterine exposure to drugs largely depends on the substance’s molecular structures and the pregnancy physiology. Drugs cross the placenta more via passive diffusion and less via active transport and pinocytosis. The placenta can metabolize drugs; furthermore, it expresses enzymes, such as cytochrome P450. The fetus also metabolizes certain drugs, especially in the final stages of pregnancy. Opioids have a rapid transplacental passage (less than 60 min), and several symptoms characterize maternal and fetal withdrawal syndrome. Opioid withdrawal significantly impacts the fetus, inducing worse outcomes and a higher risk of mortality [1,2].

Moreover, neonatal abstinence syndrome (NAS) follows delivery, lasts up to 10 weeks, and requires intensive management [3]. NAS leads to irritability, tremulousness, and temperature dysregulation, as well as to a disorganized and subsequent failure to thrive. Therefore, the prevention of NAS and its adequate management are relevant public health issues [3]. Indeed, neonatal opioid intoxication requires cooperation between the public health system, social services, and the judicial system to guarantee the health of both the mother and the baby, as well as to better implement the neonatal welfare system [3,4]. This narrative review aims to summarize the most updated evidence in the literature regarding the toxicological, clinical, and medico-legal issues of intrauterine exposure to opioids. This is conducted to suggest an evidence-based, multidisciplinary approach when dealing with such cases.

## 2. Biological Matrices

Different matrices of maternal and fetal origin are utilized to identify uterine exposure to drugs. Monitoring drug usage during pregnancy has also been done using maternal blood, oral secretions, and sweat. However, there are no standardized tests to monitor drug use with these biological matrices, which are also not widely available. Another disadvantage for these matrices, which is due to the quick elimination of most drugs, is the reduced ability to detect remote drug intakes [5]. The detection window is narrow in the case of urine, blood, oral fluids, and sweat, rendering these matrices to be not helpful in identifying sporadic use [5,6,7,8,9]. As such, testing a biological matrix with a long detection window would provide a better opportunity to identify drug exposure. The ideal situation would be to identify all drugs used during pregnancy with a single sample that is easy to collect. However, this is currently impossible.

The presence of drugs in the maternal biological matrices is not always correlated to the intrauterine exposure of the fetus to these substances because of the different degrees of placental transport of drugs. Since the uterine exposure of the fetus to drugs is not directly correlated to the presence of drugs in the biological matrices of maternal origin, due to the different degrees of placental transport of these toxic substances, it is preferable to use matrices to identify any fetal exposure to drugs in biological products from the newborn [5]. Comparing the results obtained from different biological matrices allows for improvement in interpreting the results regarding drug exposure during pregnancy. Furthermore, these matrices could be used to correlate the history of drug exposure and the potential impact on the newborn’s health [5]. However, in practical work, there are often differences between drug test results achieved by analyzing various biological matrices.

It must be noted that there are two methods of identifying drug users: self-reports or biological sample testing. Although no single approach can accurately determine the presence and quantity of drugs used during pregnancy, combining the toxicological examination with a structured interview could improve the identification of drug exposure [5,7,9,10]. Medical history derived from a self-report is a practical method for the purposes of identifying antenatal drug exposure. The only available way by which such information is obtained relates to the history of use during pregnancy and the quantity administered [5,6,7].

Unfortunately, self-reports suffer from problems resulting from a lack of information and accuracy; furthermore, pregnant women tend to underestimate or deny drug use out of fear of the consequences and the stigma associated with drug use during pregnancy [5,7]. Furthermore, an accurate history of the drugs exposure and other details are difficult to remember, thus negatively affecting the accuracy of the self-report [5,6,7,8]. The collection and the choice of the specimen in the case of a suspected intrauterine, as well as perinatal exposure to opioids, is an essential topic in forensic toxicology. Each matrix has its characteristics, advantages, and limitations, which need to be considered. Moreover, the pathologist needs to know the pharmacodynamics and pharmacokinetics of opioids to deal with a suspected case of intrauterine and neonatal opioid intoxication. In recent years, numerous authors have analyzed the best biological matrix of maternal origin, of the fetus and the newborn, in detecting intrauterine exposure to opioids. Several experimental studies have shown that the umbilical cord is an excellent matrix for the isolation of opioids, with the identification of codeine, morphine, and 6 AM. However, at the same time, they have not found identification of some synthetic opioids [9,10,11,12]. Another experimental study conducted by Colmenero et al. used different biological matrices, such as maternal hair, meconium, the umbilical cord, and the placenta, to research opioids and other drugs. Different matrices made it possible to analyze the frequency of drug use throughout the pregnancy. Maternal hair was the matrix that identified the highest number of cases and possessed the largest detection window, followed by meconium [13]. Two other scientific studies, both published in 2017, analyzed the ability of meconium, urine, and umbilical cord blood to detect intrauterine opioid exposure [14,15]; these clear findings may assist clinicians in selecting the most appropriate test to confirm a suspicion of intrauterine opioid exposure. In the following section, the properties of biological matrices are discussed.

### 2.1. Maternal Biological Matrices

#### 2.1.1. Urine

Urine is the most universally used biological matrix for the purposes of drug testing on adults. This is the case because of the non-invasiveness of sample collection and the availability of standardized tests [7,16,17]. Urine can identify recent drug use within days of the sample collection. Urine is an excellent way to identify nicotine, opiates, cocaine, and amphetamines [7,18]. On this note, screening and confirmation testing are the two main types of urine drug tests. The presence of a drug or drug class is determined from screening tests when it is higher than a predefined cutoff value. When compared to confirmation tests, screening tests typically have less sensitive and specific measurements [18,19]. Definitive testing includes gas chromatography–mass spectrometry (GC-MS), liquid chromatography–mass spectrometry (LC-MS), and liquid chromatography–tandem mass spectrometry (LC-MS/MS). Although the definitive tests possess higher sensitivity and are often more expensive than the presumptive urine drug tests, the results are more accurate and trustworthy [19]. Urine is a biological matrix used to identify fetal exposure to opioids; furthermore, methadone can be detected in the urine up to 2 weeks after the last intake, and heroin up to 72 h. In addition, morphine and codeine can also be isolated in this matrix. The main disadvantage of urine is the short detection window, as it only allows the identification of drug use in the days before collection [1,16,20,21,22].

#### 2.1.2. Hair

Maternal hair is the most sensitive biological sample in respect to detecting certain drugs during pregnancy, exposure to substances such alcohol, smoke, cocaine, opioids, cannabinoids, barbiturates, benzodiazepines, and methamphetamines, as well as to therapeutic drugs and common chemical compounds [8,20,21]. Adults’ hair grows at a rate of about 1 cm each month; thus, this must be considered when analyzing the data. Maternal hair collected during delivery may not show drug use in the days or weeks before delivery. As such, it would be advisable to postpone the collection of maternal hair to 1 month after birth. Based on the length of the hair, the specimen should be analyzed in different portions to assess the history of drug abuse [7]. However, the biases that can be derived from the adult hair test are manifold, from the color of the hair to its texture, as well as the possible inclusion of cosmetic treatments [20]. 

#### 2.1.3. Nails

The germinal matrix of the nail or the nail root under the epidermis creates layers of closely packed keratinized cells that form the nail. The newly formed keratin cells push the older cells through the cuticle, where they are differentiated (i.e., flatten and harden) to form the nail plate [5]. Adult fingernails and toenails grow distally at around 0.1 mm/d and 0.03–0.04 mm/d, respectively. However, age, sex, health status, season, environment, and exercise all affect how quickly nails can develop [5]. Maternal nail testing, based on the length of the nail that may be tested, suggests chronic exposure that may have occurred over months or weeks.

#### 2.1.4. Breast Milk

Licit and illicit drugs can range from maternal to milk circulation through to passive diffusion or carrier-mediated drug transport. The rate of drug excretion into breast milk is determined by the physicochemical properties of the drugs (such as ionizability, lipophilicity, molecular weight, volume of distribution, lipid solubility, maternal plasma protein, and lipid binding), as well as by blood flow and circulation in the mammary glands [7,22]. Drug concentrations in breast milk are influenced by the dosage, length of use, daily milk production, genotype, which may impact how medications are metabolized, and maternal health. Several scientific studies have highlighted the presence of opioids, such as morphine, codeine, and 6-AM [22], in breast milk. Breastfeeding is advised if medicines are not concentrated in the breast milk or if the exposure is not anticipated to damage the child. The Academy of Breastfeeding Medicine Clinical Protocol#21 [23] has provided breastfeeding guidelines regarding when the use of drugs is known. In respect to mothers who are receiving methadone or buprenorphine treatment, for instance, nursing is advised because of the low levels of these medicines in breast milk. Mothers who are consuming codeine should avoid breastfeeding because of the ultrarapid metabolizers that may unintentionally expose the nursing child to extremely high levels of morphine [5,20,21,22].

#### 2.1.5. Blood

Maternal blood was one of the earliest forms of biological matrix to be examined for the purposes of drug detection. For quantitative data and acute poisoning, blood is currently the best option. However, the narrow detection window and the intrusive nature of the sample collection limit the efficacy of blood tests for the long-term use of illicit drugs [8,22]. 

#### 2.1.6. Sweat

Sweat is a secretion, the production of which is stimulated by the sympathetic nervous system. This system possesses the critical task of maintaining a constant body temperature. Several mechanisms are involved in respect of the deposition of drugs in sweat, such as passive diffusion and transdermal migration. There are two ways to perform a drug test with sweat: the first involves the identification of drugs taken in the last 24 h of collection, while the second method consists of applying a dermal patch in which sweat will be collected for a period of the time variable, which is usually less than one week. Sweat is a matrix that can be quickly and easily collected. Still, it is difficult to quantify the volume of sweat expelled, thereby making this matrix useful only for qualitative tests. Several scientific studies have dealt with isolating drugs such as cocaine, cannabis, and opiates in sweat [24,25,26].

#### 2.1.7. Oral Fluid

Oral fluid is a compound tissue primarily formed by saliva. It is also where mixed gingival and buccal fluids, mucosal transudates, cellular debris, bacteria, and undigested food residues reside. The peculiarity of the oral fluid is that it better isolates the drug inside of it, instead of its metabolites [8,21]. Several factors influence the concentration of drugs in oral fluid [8,21]. The collection of oral fluid, even if it is easy to perform and not invasive, is often inadequate or insufficient. As such, there needs to be a standardized protocol. The detection window ranges from 30 min to 36 h, depending on the substance studied. Moreover, the scientific community has highlighted the presence of cocaine, cannabis, and even opiate in oral fluids [27,28,29].

The characteristics of maternal biological matrices are summarized in Table 1.

### 2.2. Neonatal Biological Matrices

#### 2.2.1. Urine

Neonatal urine is usually the matrix of choice for newborn drug testing [6,8,21,30]. The first void is frequently missed because the newborn may urinate during or immediately after delivery. Furthermore, the collection is complex, and the value of collecting and testing later voids is diminished because they are less indicative of intrauterine drug exposure [5,6,21]. However, collecting urine from newborns has produced a poor yield for the purposes of detecting drug exposure. Moreover, it only reveals maternal drug use history up to a few days before the testing. In addition, there have also been reports of analytical difficulties in regard to the different distribution of drug analytes and the composition of newborn urine from adult urine [1].

#### 2.2.2. Meconium

Meconium is a complicated, viscous substance with a dark green appearance full of metabolic waste products. It is a good choice for the purposes of determining utero drug exposures because it can potentially contain other substances to which the fetus was exposed [5,7,21,31,32]. Around the 12th week of pregnancy, meconium forms when the swallowing reflex matures; drugs go into fetal circulation directly through placental transfer and amniotic fluid ingestion, such that they are deposited in the meconium [5,6,8,20,32]. Meconium typically passes within the first one to three days of life, but in premature infants defecation may take longer [8,21]. Third-trimester exposures are more easily recognized because meconium production is nonlinear. Indeed, more than two-thirds of the meconium develops during the last eight weeks of pregnancy [5,7,8,16,20,30,32]. Meconium passage frequently takes place over several days. Meconium testing sample volumes are frequently insufficient, especially in preterm births. Additionally, the collection is often difficult because meconium is removed from a newborn’s diaper. As such, the medical practitioner must be careful not to remove milk stools or urine [5,21,32]. Numerous investigations have found that meconium possesses higher drug concentrations than other matrices, probably because drugs and their metabolites build up in meconium. Gray et al. demonstrated that an increase in the proportion of opioid-positive maternal urine samples throughout the third trimester of pregnancy was associated with opioids in the meconium, notably morphine [33]. Meconium has been proven to be more sensitive than the cord tissue or the placenta in regard to detecting buprenorphine and its metabolites in babies who have been born to women receiving buprenorphine therapy. Additionally, it is better capable of detecting cocaine or opiate re-exposure [5,7,31,34]. Although meconium has been used extensively to identify utero drug exposures, collecting the specimen is time consuming and has numerous drawbacks. Generally, drug concentrations in meconium remain stable when stored at 20 °C. The high rate of false-positive results in screening procedures, particularly immunoassay approaches, is another drawback of meconium analysis [21,31,32].

During pregnancy, the umbilical cord is a lifeline connecting the growing embryo or fetus to the placenta. It possesses two umbilical arteries and one umbilical vein, which serve as conduits for oxygen, nutrients, and waste products—such as carbon dioxide—and are shielded and protected within the Wharton jelly. It is approximately 50–70 cm (20 inches) long and 2 cm (0.75 inches) in diameter at full term. In addition, the umbilical cord is formed by the fifth week of development. Similar to meconium, this naturally complex matrix necessitates effective sample preparation techniques to reduce matrix interference [5]. The weight of tissue tested, the handling and storage of the material, the quality of the specimen submitted for testing, the recovery of drug analytes from cord tissue, and the analytical sensitivity of the methods utilized can all impact the detection window for drugs in the umbilical cord. Similar to meconium, tissue also forms nonlinearly, with the third trimester serving as the time when most of the tissue is formed. As a result, there is almost no probability that first- and second-trimester maternal drug use will be discovered [5]. The umbilical cord was studied to detect the possible fetal exposure to opioids. These studies have highlighted the presence of many opioids, such as morphine and methadone [35,36,37].

#### 2.2.3. Hair

Neonatal hair begins to grow from around 20 weeks of pregnancy and emerges on the scalp after about 3 weeks. It reflects exposure during the final trimester of a full-term pregnancy because it retains medications found in the fetal blood and amniotic fluid. The inability to identify medicines used during labor and delivery is a significant benefit of neonatal hair. However, because the neonate may have little to no hair, it is frequently impossible to gather enough material [5,9,21]. Several studies have focused on the detection of opioids in neonatal hair. Most molecules belonging to this class have been identified, such as morphine, codeine, m6g, 6-monoacetylmorphine, and methadone [38,39,40].

#### 2.2.4. Nails

Nails start to develop around 10weeks into pregnancy, and at the end of the eighth month of pregnancy (the last trimester), the neonate’s nails have grown to the tips of the fingers and toes. The neonate nail taken from the newborn represents exposure during the second and third trimesters. The disadvantages include the fact that it may be challenging to obtain enough nail samples from small newborns and that such testing is not widely available [5,21].

#### 2.2.5. Placenta

The placenta develops at about four weeks and provides the exchange of oxygen, nutrients, and waste materials between the mother and fetus [5,8]. Most pharmaceutical drugs passively diffuse across the placenta; in addition, the amount that reaches the fetal bloodstream depends on the physicochemical characteristics of the medications and their metabolites, as well as the affinity of the drug analytes for placental drug transporters. According to animal experiments with morphine, methadone, and meperidine, opiates readily penetrate the placenta and reach their peak blood levels in the fetus soon after intravenous injection [5,8,22,41,42,43,44]. Based on its high level of lipid solubility, heroin has a high index of prenatal exposure, since it easily crosses both the blood–brain barrier and the placenta. De Castro et al. created a technique in 2009 for the purposes of measuring heroin metabolites in the placenta. Morphine was discovered, when analyzing the placentas of five pregnant opioid-dependent women, in one sample at a concentration of 41.3 ng/mg. However, 6-AM was not found, thereby rendering it impossible to detect heroin use [45]. In pregnant women receiving buprenorphine maintenance treatment, buprenorphine glucuronide concentrations in the placenta were significantly correlated with the maternal daily dose, according to Concheiro et al. [46]. The maximal NAS score and the length of the infant were both positively and adversely linked with the norbu-prenorphine/norbuprenorphine glucuronide ratio. Placenta norbuprenorphine glucuronide concentrations were also positively correlated with the time to NAS onset and negatively correlated with the duration of NAS. Moreover, buprenorphine was less accurate at predicting neonatal outcomes than was norbuprenorphine glucuronide at higher concentrations [46,47].

#### 2.2.6. Vernix

The creamy-white film that covers the baby’s skin throughout the last trimester of pregnancy and is often present on the newborn’s skin at birth is known as the vernix caseosa, which is made up of a mixture of water (80.5%), protein (10%), and a fat (10%) called vernix. Although the quantity of samples available for testing varies, it is easily collected from a newborn’s skin following delivery by swabbing it with gauze [5,21].

#### 2.2.7. Amniotic Fluid

The amniotic fluid in the early stages of pregnancy comprises a filtrate of fetal cells and maternal blood. Furthermore, as the fetus grows, it gradually changes with gestational age. The fluid is similar to the fetal plasma that is found between weeks 10 and 20 of gestation; in addition, toward the second half of pregnancy, it is primarily made up of fetal secretions, such as lung fluids and urine. An amniocentesis operation is used to collect amniotic fluid; this method is intrusive and may harm the unborn child [5,8,21]. The amniotic fluid is continuously swallowed, such that the fetus may again be exposed to medicines passed on through the urine [5,8,21]. The properties of neonatal biological matrices are summarized in Table 2.

## 3. Analytical Issues

### 3.1. Preanalytical Phase

The physicochemical characteristics of the analytes and the complexity of the sample tissue or fluid from which they are to be extracted dictate the method that can be used for sample preparation [20,21]. It takes a great deal of preanalytical processing to homogenize, digest, or otherwise prepare newborn specimens that are not liquid (such as meconium and tissue) for analysis. Further preanalytical processing (such as hydrolysis and derivatization) may be carried out to reduce analytical interferences and increase the possibility of identifying the desired analyte. These specimens’ analytes are typically separated and purified using certain techniques, such as liquid–liquid extraction (LLE), solid-phase extraction (SPE), and solid-phase microextraction (SPME) [5,22].

These procedures frequently entail washing processes—i.e., an acid, base, or enzymatic hydrolysis—and lengthy incubation times [7]. The extraction of drug analytes from the intricate matrix is one of the most challenging analytical problems in regard to analyzing solid specimens. For the purposes of mass spectrometric techniques, removing lipids and proteins is crucial as they could cause ion suppression and limit the detection of drug analytes [5]. Several methods, such as liquid–liquid extraction, solid-phase extraction, and supported liquid extraction, have all been used to extract drugs from meconium or umbilical cord tissue. The recovery of the drug from the matrix and the technique’s sensitivity directly impacts the window of detection that is attained, such that the significance of the extraction should not be understated. Furthermore, extraction may result in the drug analytes becoming lost. In addition, it may not be uniform across all the elements of a multianalyte panel. Furthermore, how temperature affects different matrices over time and how the long-term stability of drugs and metabolites vary may need to be better understood. Positive results need to be verified using gas chromatography/mass spectrometry because immunoassay is a relatively generic test. Additionally, opioid abuse is not always linked to opioid presence confirmation [7,8,16]. Alternative causes include passive drug exposure, consuming tainted food or drink, or taking prescription drugs that either contain the drug or are converted into it [5,6,7,8].

### 3.2. Screening Test

Enzyme multiplied immunoassay (EMIT), fluorescence polarization immunoassay (FPI), radioimmunoassay (RIA), or ELISA 2-6 are all frequently used for screening urine or meconium. 

When using class-based immunoassays, point-of-collection/point-of-care test cups, dipstick-type strips, or automated instrumentation, hospital laboratories routinely do urine drug screening onsite. 

These tests are usually performed on automatic platforms and employ antibodies that have reactivity to ward several medications in the same class; furthermore, opioid immunoassays detect the presence of codeine, morphine, hydrocodone, and hydromorphone1,2-6. However, when compared to confirmation methods, screening assays typically have the following drawbacks: limited specificity, low sensitivity, and low reactivity in respect to specific medicines within a pharmacological class. The most commonly used screening immunoassays for opioid detection are the FPI and EMIT [30,39,40,48,49,50,51,52].

### 3.3. Confirmation Methods

Confirmation techniques typically use either gas chromatography (GC) or liquid chromatography (LC) to separate compounds within the matrix, followed by MS for detecting and quantifying individual drug analytes. Confirmation techniques have higher sensitivity and specificity; furthermore, they are based on a different analytical principle. Due to their well-documented capacity to generate sensitive and accurate results for the purposes of drug testing, GC-MS, liquid chromatography-tandem mass spectrometry (LC-MS/MS), and liquid chromatography time-of-flight mass spectrometry (LC-TOF-MS) are all widely used for the purposes of quantitative and/or qualitative analysis [5,21,30,47,48,49,50].

Vinner et al. investigated the gestational profile of opiate exposure. They achieved this thanks to the toxicological analysis of three different matrices: maternal and neonatal urine, as well as hair and meconium using FPI and EMIT as the screening immunoassays, and GC-MS as a confirmatory method [47]. The confirmation with GC-MS for the samples resulted in a positive for the immunoassay, which allowed us to determine different opioid metabolites, such as morphine, codeine, and 6-monoacetylmorphine [51,52,53]. Additionally, the analysis of meconium via GC-MS can open a wide window for the detection of fetal exposure to cocaine and opiates [45,54].

The analytical issues of the revised papers are summarized in Table 3.

## 4. Clinical Issues

Over the past 10 years, opiate use during pregnancy has drastically increased and is now considered to be a serious public health issue. Prescription opioids, illicit opioids, and opioid replacement therapy are all being used by more women. According to Walsh SL et al., they issued an increase in opioid use in pregnant women because of the widespread use of acute/chronic pain treatment during pregnancy [55]. 

In fact, patients may receive opioid prescriptions during pregnancy for untreated opioid-use disorder, opioid abuse, or persistent pain or addiction. In addition, as Casper et al. state, mixing benzodiazepines, alcohol, or nicotine is a practice that is fairly widespread [56]. Opioids have been utilized in maintenance therapy for heroin addicts since the 1960s, but heroin substitutes are now being administered [57]. 

Opioid maintenance therapy is practical for at least three reasons, according to the American College of Obstetricians and Gynecologists: it decreases the mother’s risk of relapsing, reduces continued high-risk activity, and improves perinatal outcomes by preventing frequent withdrawal during pregnancy [58]. Opioid maintenance therapy regimens have traditionally relied heavily on methadone, while buprenorphine use has recently increased. Pregnant women have different methadone pharmacokinetics from the general population and these pharmacokinetics can alter dramatically over the course of the pregnancy [59]. For instance, as stated by Megan W. Stover et al., among pregnant women, the half-life of methadone, from an average of 22–24 h, is reduced to 8 h. Even though methadone is typically administered daily, split-dosing (every 12 h) can be used to account for increased clearance during pregnancy [3]. Buprenorphine is a more recent alternative to opiate maintenance therapy in pregnancy. It is a partial opioid agonist approved in 2002 for the medication-assisted treatment of opiate dependence [60].

According to Jones HE et al., Buprenorphine has been shown to be superior to methadone in several ways for the management of NAS, including a lower risk of overdose (caused by reduced intrinsic receptor efficacies), less-abrupt withdrawal, fewer drug interactions, and easier access to prescriptions [61].

The abrupt cessation of fetal exposure to substances that the mother consumed or abused while she was pregnant resulted in the formation of the NAS. The term NAS is used to describe withdrawal from substance exposure; the term neonatal opioid withdrawal syndrome (NOWS) refers to the symptoms and signs that are specifically due to opioid withdrawal. Newborns exposed to opioids in utero may develop neonatal opioid withdrawal syndrome (NOWS), lower birth weight, smaller head circumference, and a higher risk of sudden infant death syndrome [62].

NAS is a multidistrict systemic disorder. The signs of neonatal abstinence have classically been divided into four major categories: involving central nervous system, gastrointestinal system, respiratory system, and autonomic nervous system. Even though NOWS seldom results in death, it can lead to significant illness and frequently necessitates prolonged hospital stays. Depending on the kind and quantity of substance consumed, the severity of this pathology’s symptoms may vary [61,62,63].

When compared to methadone, buprenorphine sometimes decreases in the frequency and gravity of NOWS, according to newly available research. Buprenorphine has a number of drawbacks, including high dropout rates, challenging treatment start-up, a higher risk of drug diversion, possible hepatic side effects, and lack of long-term data regarding safety during pregnancy and in young children [63]. 

According to the Maternal Opiate Treatment Human Experimental Research’s findings, pregnant women who received buprenorphine treatment had less-severe cases of NOWS and required shorter stays in hospitals than those mothers who received methadone treatment; despite this, limited evidence is available to determine the best pharmacological agents to help with maternal opioid abstinence [64]. 

It is widely known that the human placenta controls how chemicals and nutrients are transferred to the fetus. Drugs, pharmaceuticals, and their metabolites can pass through the placenta and into the bloodstream of the fetus with ease. A few factors that affect this placental transfer include the specific drug, the amounts of the drug in the mother’s and fetus’s circulations, the way and when it is administered, the mother’s and fetus’s genetic makeup, and the co-administration of other medications [65]. As opioids are used more often to treat chronic benign conditions, more infants are being treated for side effects from intrauterine opioid exposure. The naturally occurring opioid morphine has been the subject of almost all prenatal exposure investigations, but since 2014, there has been a 300% increase in interactions with synthetic opioids, especially fentanyl, which is 50–100 times more powerful than morphine [66,67]. Alipio et al. found that perinatal fentanyl exposure results in neurobiological deficits that last until adolescence. The effects of this exposure include the suppression of adaptation to sensory stimuli, impairment of synaptic transmission in the S1 and ACC, suppression of cortical oscillations, abnormal dendritic morphology of cortical pyramidal neurons, and altered mRNA expression of genes that regulate synaptic transmission and dendritic morphology [67]. Due to the widespread use of synthetic opioids, especially in Western countries [68], further research should focus on determining the short- and long-term effects on newborn caused by intrauterine exposure to these substances.

Healthy infants may find the shift to extrauterine life stressful; however, the adjustment is often significantly more difficult for newborns exposed to drugs while still in the womb. Several literature studies state that opioid exposure during pregnancy also greatly raises the risk of preeclampsia, stillbirth, preterm, and sudden infant death syndrome (SIDS) [64,65,69]. As suggested by Nicole A. Bailey et al., there may be a link between prenatal opioid usage and congenital defects, such spina bifida, gastroschisis, and congenital heart disease. This assertion, however, was not supported by a recent comprehensive analysis of case-control and cohort studies on the topic [69].

The first case of a neonate who manifested opioid withdrawal signs was documented in 1875, but only in 1903 was the first case successfully treated, and it was referred to as congenital morphinism in the early 1900s and is the most frequent consequence of utero opioid exposure [70]. Congenital morphinism was termed NAS by Dr. Loretta Finnegan in the 1970s [71]. Even while other drugs—including benzodiazepines, amphetamines, cocaine, and barbiturates—can cause NAS(as revealed within the study of Krans EE et al.), babies who have been exposed to opioids are more likely to develop it [71]. While opiate maintenance therapy lessens a number of unfavorable pregnancy outcomes, it does not stop the emergence of NAS [72]. All newborns exposed to opiates in utero should be closely monitored for the development of NAS/NOWS because there is a 60–80% chance that they will develop this severe illness [64], which is a finding that is in agreement with Johnson et al.

The NAS/NOWS condition, which is intricate and extremely variable, affects the newborn when the placenta is separated from the fetus at birth. It is distinguished by gastrointestinal difficulties, autonomic nervous system dysfunction, and hyperirritability of the central nervous system [73]. Excessive impatience, bad sleep, stronger muscles, tremors, and skin excoriations caused by excessive movement, overheating, diarrhea, excessive sleepiness and sweating, stuffy nose, and sneezing are all among the most frequent symptoms. Additionally, 2–11% of newborns with NAS may experience seizures. According to the findings of Seib CA et al., newborns exposed to opiates show significant variance in the timing and presentation of symptoms [74]. The causes of this variability are unclear and probably multifaceted in nature. Examples of possible causes include differences in maternal treatment, abnormalities in placental opioid metabolism, pharmacogenomics, and neonatal comorbidities, to name a few. The signs of NOWS frequently occur between 24–48 h after delivery, 36–60 h for buprenorphine and 48–72 h for methadone, depending on the prior maternal dose (but up to 5 days because of the long half-life) [75]. 

As claimed by Chasnoff IJ et al., drug exposures in the past, such as from using benzodiazepines, anti-depressive medications, or smoking cigarettes, may change the development of symptoms and worsen NAS [76]. The most prevalent form of evaluation (often conducted with modifications) is the Finnegan scoring system, which can identify which newborns need pharmacologic therapy. Every 3 to 4 h, a 31-item scale from the classic Finnegan scoring system is used to evaluate the prevalence and severity of different NAS-related symptoms. Every assessment should consider the conduct that was seen throughout the preceding three to four hours. It should be noted that the Finnegan scoring system has a high intra-observer variability and is specifically intended for term newborns.

Non-pharmacologic therapy is the first line of defense in the treatment of NOWS. Usually, frequent hypercaloric meals are provided to encourage growth and reduce hunger. The care of the newborn by the mother is a crucial aspect of non-pharmacologic therapy. It is believed that non-pharmacologic care of infants with NOWS deserves more attention in the care of newborns. To develop their capacity for self-regulation, newborns and caregivers must continually alter their physiological and behavioral responses. This process is known as “co-regulation”, which depends on continuing experiences of both. First, giving the dyad a secure living space and supportive environment is crucial. Rooming in and the environment for a newborn with NOWS during and after the hospitalization can provide neuroprotection for a brain that is sensitive, dysregulated, and growing quickly [77].

Furthermore, the majority of newborns with NOWS require pharmacologic treatment [78]. Opioid substances (such as morphine and methadone) are generally considered more effective than other medications in treating NOWS. However, a Cochrane review released in 2010 concluded that there was not enough proof to recommend one opioid over other sedatives (phenobarbitone or diazepam) or other supportive treatments (swaddling, relaxation baths, settling, or massage). However, the use of opiates raised the treatment efficacy compared to diazepam [79]. The most suggested first-line treatment is morphine or a diluted tincture of opium taken orally [79]. 

Since methadone has a longer (and more variable) half-life and needs less-frequent administration and titration, it can be used instead of morphine. It is also currently being investigated as a possible drug that could be used to treat NOWS with sublingual buprenorphine [80]. 

Every 3–4 h, a 31-item scale from the classic Finnegan scoring system is suggested for the evaluation of the presence and severity of various NAS-related symptoms [3]. The entire 3 to 4 h preceding the exam should be considered in each evaluation. It should be noted that the Finnegan scoring system has high intra-observer variability and is mainly designed for term newborns. The maximum Finnegan score in regard to the infant’s weight, or a combination of the two, is used to determine the dosage of these drugs. Second-line medications, such as phenobarbital and clonidine, are used when symptoms are still not under proper control on the highest dose of treatment. Once symptoms have been stable for 24–48 h, the tapering of pharmacologic treatment often starts [81]. Infants and children who were exposed to opioids in utero have been reported to experience negative neurodevelopmental consequences. However, the information on long-term neurodevelopmental function is scarce [82]. 

As stated by Megan W. and Stover et al., NOWS occurs less frequently in preterm newborns than in term babies for a variety of reasons, including the fetal CNS’s immaturity, lower cumulative drug exposure, less placental transfer, delayed hepatic and placental metabolism, and a reduced drug deposition that is due to the lower fat content. It should be noted that the absence of a corroborate scoring system created specifically for these people restricts the ability to assess NAS/NOWS in preterm infants [3]. In comparison to children born without NOWS, recent research suggests that infants with NOWS are often more prone to experience developmental delays or speech or language impairments [83]. The key points of NOWS identification are summarized in Table 4.

Bradley S. and Peterson et al. found that inborn measurements of brain anatomy, tissue architecture, and metabolites showed a direct relationship with prenatal illegal drug exposure [84]. Drug-exposed newborns, particularly those who have been exposed to heroin or methadone, had smaller head sizes overall, smaller brain volumes, and lower cognitive abilities [85]. Children who have been exposed to opioids are generally more prone to exhibit disruptive behavior, attention deficit issues, and the need for thorough psychiatric treatment. 

In agreement with Honein MA et al., a longitudinal study of children exposed to opioids during pregnancy may increase our understanding of the potential teratogenic effects of opioid use, such as the confounding effects of exposure to other substances during pregnancy (such as alcohol), as well as environmental and psychosocial factors [86].

In a recent assessment of the topic of opioid use during pregnancy, healthcare professionals should routinely assess all pregnant women for drug use through history and physical examinations, as well as with proven screening tools [87]. Therefore, it is crucial that individuals who provide maternal care try to identify women who use drugs and direct them toward treatment choices.

According to the American Society of Addiction, a urine screening test to identify drug abuse should be conducted according to state regulations, which vary by state of residence, with the patient’s permission, and to confirm reported or suspected drug usage [88]. Neonatal toxicology testing should start as soon as a baby is born to a mother who has used drugs previously or is suspected of doing so. All delivery staff members must be aware of drug use to help the neonate adapt to extrauterine life and provide neonatal or pediatric support.

In correspondence with the findings of Wong S. et al., the midwife may be tasked with ordering neonatal toxicological tests in the role of the intrapartum healthcare practitioner. If the collection takes place along with the first two emissions of urine, newborn pee analysis can identify recent maternal drug use [89]. As meconium toxicology tests show drug usage as early as the second trimester, the data provided a more detailed description of drug use by the pregnant mother. Umbilical blood collection is less invasive and more valuable than urine or meconium collection and is a possible third technique used for newborn toxicity testing. Finally, another form of toxicity screening involves the utilization of maternal and neonatal hair.

When a newborn toxicology test yields a positive result, healthcare professionals must take into consideration the fact that the baby had drug exposure during the pregnancy and is thus at risk for NOWS. The Finnegan neonatal abstinence scoring tool (FNAST) may be used by neonatal caregivers to thoroughly and objectively evaluate the newborn’s withdrawal signs and symptoms. The scoring of the FNAST should start two hours after birth and should continue every three to four hours while the infant is receiving care. The FNAST extends for at least 48 to 72 h after withdrawal agents are stopped and includes all NOWS signs and symptoms experienced during withdrawal and management [90]. 

The eat, sleep, and console (ESC) approach, which was recently introduced and was first published by Grossman et al. in 2017, has introduced a different paradigm that emphasizes nonpharmacologic management of infants’ symptoms and offers a framework for starting treatment based on functional impairment [91]. The clinical management of newborn opioid withdrawal syndrome is based on the ESC model (NOWS). The Finnegan scoring system and ESC system were compared in the study conducted by Kelsey Ryan et al. They found that ESC scores correlated with components of the Finnegan score system that predict the severity of NOWS; on the other hand, the ESC system did not associate with elements of the Finnegan score that do not predict the severity of NOWS. They also suggest that transitioning from the Finnegan score to the ESC system could reduce hospitalization and dependency on pharmacologic treatment for newborns affected by NOWS [92]. 

A neonatologist must be informed of the circumstances to adequately evaluate the child and perhaps even transfer care to a neonatal unit that is equipped to help a newborn with NOWS if the neonatal team is not currently caring for the baby. Infants and children exposed to opioids in utero have been reported to experience negative neurodevelopmental consequences. However, as most studies are small and cannot distinguish between the effects of in utero exposures, postnatal treatments, and environmental variables, there is a shortage of information addressing long-term neurodevelopmental function. Children exposed to opioids are more likely to exhibit disruptive behavior, attention deficit issues, and the requirement for a thorough psychiatric referral. Long-term follow-up is, for this reason, a relevant public health issue.

## 5. Medico-Legal Issues

Pregnancy-related opiate misuse carries a number of dangers, most of which are connected to the consequences of withdrawal for the mother and her fetus, or the concurrent hazards of any associated behaviors. It may be challenging for a pregnant mother to abruptly stop using opioids. Others may rely on drugs such as methadone to stop drug relapse during pregnancy [93]. A drug should be stopped if it is considered unsafe for both the mother and the fetus. In agreement with what the American Pregnancy Association states, any substance taken while pregnant must be viewed as potentially dangerous to the fetus; in addition, the risks versus the benefits of its use should be carefully weighed [94]. Furthermore, abusing these drugs puts the health of the developing child, the neonate, and the fetus at risk. As such, this represents a serious public health problem [95].

As reported by Megan T. Frey et al., many female opioid abusers engage in polysubstance misuse, frequently in an effort to treat an underlying mental health condition or alleviate withdrawal symptoms with more accessible drugs [96]. Women who use substances are more likely to self-report conditions like high poverty rates, intimate partner violence, a history of physical or sexual abuse, post-traumatic stress disorder, and mental illness [97]. Although there has been a simultaneous rise in both child welfare cases and opioid use disorders, according to Korry et al., the two developments cannot be directly connected at this time because of data limitations [98]. 

Local child welfare institutions state that the opioid epidemic can be blamed for the recent uptick in incidents of neglect and abuse [99]. Due to concerns about losing their children to child protection service investigations, as a study by Falletta et al. observed, some mothers were not allowed to receive treatment or were delayed, while other pregnant women avoided receiving treatment in order not to undergo drug tests [99]. 

According to the National Institute on Drug Abuse, addiction is a chronic condition that can be successfully managed and treated like other chronic disease processes. Social support, the quality of the patient–provider relationship, and access to therapy are all necessary to successfully treat substance-use disorders. In accordance with the ethical rule of nonmaleficence, doctors must refrain from employing humiliation or unfavorable criticism to persuade women to seek or continue receiving care. Pregnant addicts experience humiliation and criticism on top of their already high personal and societal obligations because they appear to go against the conventional moral expectation that pregnant women act in the best interest of the fetus. Criminalizing women for possessing chronic health issues while pregnant is unethical from a medical and moral standpoint and feeds societal stigmas. Regardless of existence, the right to be born healthy and protected, including throughout the intrauterine phase, must be recognized, whether interpreted as a subject or as a person, based on the many interpretations.

The implementation of integrated care systems that offer medical treatment, social services, and mental support has resulted in a great decrease in substance usage and relapse [96,97,98,99].

When faced with the suspicion that a child may suffer or risk suffering injuries, physical or mental, handicap, or pathological conditions that highlight a condition of abuse or negligence, health professionals must report the case in question. According to the Italian jurisdiction, the patient must consent before any medical procedure. If the mother does not want to agree to have blood or urine taken, these tests cannot be conducted. In the case of a minor, especially a newborn, who cannot yet act, the parents must provide consent to proceed with the medical procedures. However, suppose the doctor decides that specific investigations are required because he believes the child’s life may be at risk because of negligence or drug misuse. In that case, he must contact the appropriate judicial authority. In all healthcare settings worldwide, mainly where a high rate of opioid abuse is observed during pregnancy, standard protocol and screening and clinical testing strategies should be implemented to anticipate diagnosis and point out further actions in cooperation with social services and judicial authorities to protect and guarantee adequate health and assistance for both the mother and the newborn [100]. 

To inform the family about the best ways to care for the infant, the mother’s healthcare professional should collaborate with neonatal care specialists. The consultation of social services guarantees that the newborn’s post-hospital care is adequate and suitable in accordance with the Child Abuse Prevention and Treatment Act. It is imperative to motivate women who are opioid dependent to seek out and keep up with medical care. As a result, it will be important to test not only those women who voluntarily admit to abusing opioids but also those who have medical disorders and where doctors note the risk of abuse or neonatal suffering.

A study conducted by Green et al. states that children of substance-using mothers who finish at a minimum one recovery period spend a shorter time in protective custody and are successfully reunited with their parents more frequently [101]. Although it can be challenging to obtain Child Protection Services (CPS) clients for participation in substance-misuse treatment, according to Taplin et al., half of the women who enroll in these programs are mothers of dependent children, and one-third of these have lost parental rights [102]. Knowing how other social institutions, such as the criminal justice system, can either facilitate or obstruct treatment may provide one with a more comprehensive viewpoint on the best way to include opioid abusing in the child welfare system. Supplementary studies are required to better understand how child protection services and drug misuse treatment programs interact and what effects they have on individual results.

To give clarity and direction to policymakers at the national and local levels, it is crucial to collaborate and coordinate guidelines, advocacy positions, and research projects involving prenatal substance use and NAS. This literature review highlights the necessity of a multidisciplinary approach in cases of neonatal opioid intoxication. Cooperation between different professional figures is, in fact, crucial to substantially impacting the critical public health issue confronting our vulnerable population [1,103,104,105]. The gynecologist has the role of following the regular course of pregnancy, identifying the risk conditions, and promoting the well-being of the fetus and mother. The pediatrician and the neonatologist must clinically identify the clinical factors suggesting an intoxication or a neonatal abstinence syndrome, thereby ensuring the newborn’s health and initiating the process of protection, welfare, and judicial investigations. The role of the forensic toxicologist is essential for the purposes of detecting exposure to opioids, thereby choosing the appropriate matrix to be used and providing laboratory elements on which to base clinical, social, and forensic options [2,106,107]. A critical public health measure could consist of establishing territorial referral centers for these conditions to guarantee the presence of specialized personnel to recognize and identify neonatal opioid intoxication and NAS/NOWS. Research and development are urgently needed to improve the identification, care, and protection of high-risk neonates as the number of births impacted by maternal opiate dependency keeps growing.

## 6. Conclusions

Protecting newborns and mothers requires a standardized method to detect opioid use and exposure during and after pregnancy. The choice of the appropriate specimen to analyze for the purpose of detecting in utero drug exposure will depend on the availability of the specimen, as well as on specific clinical and forensic issues. Each specimen has advantages and limitations. Urine or meconium screening of the newborn typically provides essential information to clarify intrauterine and perinatal opioid exposure. Although urine screening is simple to administer, it has the drawback of only identifying recent exposures. The benefit of meconium testing is that it can screen for drug exposure going back as far as 20 weeks of gestation. Further research is required to standardize testing and to better understand the distribution of opioid derivatives in each specimen type, as well as the clinically relevant cutoff concentrations in quantitative testing results. To best care for pregnant women with opioid disorders, the fetus, and the neonate following birth, healthcare personnel must get training that promotes multidisciplinary care and cuts across barriers between specialized areas. To implement social welfare and ensure that the baby has appropriate custody when necessary, a multidisciplinary approach is required, involving the collaboration of obstetricians, pediatricians, nurses, forensic physicians and toxicologists, social workers, addiction specialists, and politicians. The healthcare system should encourage multidisciplinary activity in this field and direct suspected maternal and neonatal opioid intoxication cases to local referral centers.

## Figures and Tables

**Table 1:** The properties of maternal biological matrices summarized.

Maternal Biological Matrices	Advantages	Disadvantages	Detection Window
URINE	Represents the most-used matrix; The collection is easy and non-invasive.	Restricted detection window; Easily adulterate.	Few days.
HAIR	The collection is not invasive and is easy to carry out; It is a very stable matrix (even for years); It has turned out to be more sensitive than the other matrices.	Unable to detect recent drug use; Possible biases derived from the color of the hair; The detection of drugs depends on the length of the hair.	One year maximum.
NAIL	The collection is simple and non-invasive; Long detection window;	Sebum and sweat can contaminate the sample; The detection of drugs depends on their length.	Few weeks.
BREAST MILK	The collection is easy and non-invasive; Reflects postpartum exposure.	The collection can be performed only in women who are breastfeeding; High variability of proteins and lipids, which makes interpretation of results difficult; It is a matrix that changes during breastfeeding.	Few days.
BLOOD	It is one of the most commonly used matrices;	The collection is invasive and requires qualified personnel.	Few days.
SWEAT	The collection is easy and non-invasive; Longer urine detection window;	Harvesting can cause skin irritation; Individual variations within sweat production; Estimating the volume of sweat produced is complicated.	Few days.
ORAL FLUID	Sample collection is simple, fast, and non-invasive; Availability of devices as collection points;	It is common to collect an inadequate sample volume; The collection procedure is not standardized; Possible unintentional contamination.	Few days.

**Table 2:** The properties of neonatal biological matrices summarized.

Neonatal Biological Matrices	Advantages	Disadvantages	Detection Window
URINE	Specimen of choice for the purposes of newborn drug testing.	The first void is frequently missed.	Few days.
MECONIUM	The collection is not invasive; Detects drug exposure for the second and third trimesters.	This matrix is only available a few days after delivery; Easily contaminated by urine or milk stool; Identifies drugs administered during labor and delivery; Prolonged storage can alter the stability of the drugs.	Second and third trimester of pregnancy.
UMBILICAL CORD (tissue or blood)	The collection is easily carried out and done so in a single time; It does not identify the drugs taken after birth.	Identifies the medications taken during labor and delivery; Maternal blood can contaminate this matrix.	Third trimester of pregnancy.
HAIR	The sample can be stored at environmental temperature; It reflects drug exposure in the third trimester of pregnancy; Avoids the detection of drugs administered during labor and delivery.	It may be difficult or impossible to obtain; Inability to detect recent drug use; The detection of drugs depends on the length of the hair.	Few months.
NAIL	Neonatal nail collected at birth accounts for second and third trimester exposure; Avoids the detection of drugs administered after birth.	It may be challenging to obtain enough nail samples from small newborns; The test is not widely available.	Few weeks.
PLACENTA	Easy and noninvasive collection; Avoids the detection of drugs administered after birth.	Requires additional sample preparation and efficient cleanup; The test is not widely available.	Few days.
VERNIX	Easy and noninvasive collection; Sample can be easily stored until analysis.	May be contaminated with urine or milk stool; Drugs administered during labor and delivery may be detected	Last 24 weeks of gestation.
AMNIOTIC FLUID	Requires minimal sample cleanup.	Risk of possible complications is associated with collection procedure; Sampling procedure is highly invasive.	Few months.

**Table 3:** A summary of the analytical methods to detect opioids in maternal and neonatal matrices.

Biological Matrices	Authors, Year	Sample Preparation	Extraction–Separation	Analytical Methods	Opioids Identified
MECONIUM	Concheiro, 2017 [30]	///	LLE	FPIA-EMIT/GC-MS	COD-MOR-6AM-METH
	Xavier Joya, 2016 [35]	Methanol	SPE	LC-MS	COD-MOR-6AM-METH
	Lozano, 2007 [39]	///	SPE	LC-MS	6AM-MOR-COD
	Vinner, 2003 [47]	///	LLE	FPIA-EMIT/GC-MS	6AM-MOR-COD–
	Marin, 2016 [49]	Methanol	SPE	LC-MS e HPLC	COD-MOR-6AM-METH
	Kintz, 1993 [51]	///	LLE	FPIA-EMIT/GC-MS	COD-MOR-6AM
	Pichini, 2003 [54]	///	SPE	LC-MS	NBUP
UMBILICAL CORD	Xavier Joya, 2016 [35]	Formic acid	SPE	LC-MS	COD-MOR-6AM-METH
	Concheiro, 2013 [36]	Acetyl nitrile	SPE	LC-MS	COD-MOR-6AM
	Stolk, 1997 [37]	Formic acid	SPE	LC-MS	MOR-METH
NEONATAL HAIR	Marchei, 2006 [38]	///	LLE	GC-MS	6AM-MOR-COD-METH
	Lozano, 2007 [39]	///	///	FPIA-EMIT/GC-MS	6AM-MOR-COD-METH
	Ostrea, 1980 [40]	///	///	FPIA-EMIT/GC-MS	COD-MOR-6AM-METH
NEONATAL URINE	Vinner, 2003 [47]	///	LLE	FPIA-EMIT/GC-MS	6AM-MOR-COD
	Kintz, 1993 [51]	///	LLE	FPIA-EMIT/GC-MS	COD-MOR-6AM
MATERNAL HAIR	Kintz, 1993 [51]	///	LLE	ELISA	///
MATERNAL URINE	Falcon, 2010 [16]	///	///	LC-MS	COD-MOR-6AM
	Vinner, 2003 [47]	///	LLE	FPIA-EMIT/GC-MS	METH
	Kintz, 1993 [51]	///	LLE	FPIA-EMIT/GC-MS	6AM-MOR-COD
MATERNAL BLOOD	Falcon, 2010 [16]	///	///	GC-MS	COD-MOR-6AM
BREAST MILK	Falcon, 2010 [16]	///	SPE	LC-MS-MS	COD-MOR-6AM

LLE: liquid–liquid extraction; SPE: solid-phase extraction; FPIA: fluorescence polarization immunoassay; EMIT: enzyme-multiplied immunoassay; GC-MS: gas chromatography–mass spectrometry; LC-MS: liquid chromatography–mass spectrometry; HPLC: high-performance liquid chromatography; ELISA: enzyme-linked immunosorbent assay; LC-MS-MS: liquid chromatography–tandem mass spectrometry; COD: codeine; MOR: morphine; 6AM: 6 acetyl morphine; METH: methadone; and NBUP: buprenorphine.

**Table 4:** A summary of the key points for the identification of NOWS.

Neonatal Opioid Withdrawal Syndrome (NOWS)	
Predictive factors	Maternal opiate dose; Maternal maintenance agent; Exposure to additional substances; Gestational age.
Timing of onset of symptoms	For heroin: 24–48 h of life; For buprenorphine: 36–60 h of life; For methadone: 48–72 h of life.
Symptoms	Hyperirritability; Autonomic nervous system dysfunction; Seizures; Irritability; Poor sleep; Hyperthermia; Sweating; Sneezing.
Long-term outcomes	Attention deficit disorders; Disruptive behavior; Smaller brain; Thinner cortex; Reduced cognitive ability.
Assessment	4–7 days of inpatient monitoring; Finnegan score every 3–4 h.
Non-pharmacologic treatment	Gentle, soothing environment; Hypercaloric feeds; Maternal care.
Pharmacologic treatment	Oral morphine; Methadone; Phenobarbital; Clonidine.

## Data Availability

Data sharing is not applicable; no new data were created or analyzed.
